# Venous thromboembolism in cancer patients: an underestimated major health problem

**DOI:** 10.1186/s12957-015-0592-8

**Published:** 2015-06-20

**Authors:** Jihane Khalil, Badr Bensaid, Hanan Elkacemi, Mohamed Afif, Younes Bensaid, Tayeb Kebdani, Noureddine Benjaafar

**Affiliations:** Radiation Oncology Department, National Institute of Oncology, Hay Riad, Rabat, 10000 Morocco; Vascular Surgery Department, Ibn Sina Hospital, Souissi, Rabat, 10000 Morocco

**Keywords:** Venous thromboembolism, Cancer, Thromboprophylaxis, Anticoagulation

## Abstract

Venous thromboembolism (VTE) is a major health problem among patients with cancer, its incidence in this particular population is widely increasing. Although VTE is associated with high rates of mortality and morbidity in cancer patients, its severity is still underestimated by many oncologists. Thromboprophylaxis of VTE now considered as a standard of care is still not prescribed in many institutions; the appropriate treatment of an established VTE is not yet well known by many physicians and nurses in the cancer field. Patients are also not well informed about VTE and its consequences. Many studies and meta-analyses have addressed this question so have many guidelines that dedicated a whole chapter to clarify and expose different treatment strategies adapted to this particular population. There is a general belief that the prevention and treatment of VTE cannot be optimized without a complete awareness by oncologists and patients. The aim of this article is to make VTE a more clear and understood subject.

## Review

### Introduction

Cancer is recognized as an independent and major risk factor for venous thromboembolism (VTE) [1,2]. According to available data and to population-based studies, cancer is in fact associated with a 4.1-fold greater risk of thrombosis [3,4]. Also, VTE is associated with a high potential of morbidity and mortality in cancer patients [5,6] it is indeed the second leading cause of death in cancer patients [7]. Occurrence of VTE has been proven to increase the likelihood of death in cancer patients by two- to sixfold [5,7].

The association between cancer and thromboembolism was first reported by Trousseau in the nineteenth century [8], since the awareness of the impact of thrombotic complications in cancer patients and the need for early management and prophylaxis is increasing. In our review, we have found more than 30 practice guidelines and major reviews on cancer and VTE [9-14].

In contrast, recent surveys have noted low compliance rates and an underutilization of prophylaxis in hospitalized cancer patients [15-18].

In current oncology practice, management and prevention of VTE is frequently encountered; the most challenging part will be to improve the awareness of the need of an early detection and management of VTE before dealing with mortal complications.

In our review, we tried to encompass the most published guidelines, meta-analyses, systematic reviews, and international articles relevant to venous thromboembolism risks prophylaxis, and its management in cancer patients.

### Methods

To proceed with the review, we electronically searched the following databases: the Cochrane Central Register of Controlled Trials (CENTRAL), MEDLINE (1966 onward; accessed via Ovid), EMBASE (1980 onward; accessed via Ovid), and ISI Web of Science (February 2010). The search strategies combined terms relating to venous thromboembolism, pulmonary embolism, cancer, screening, diagnosis, management, anticoagulants, prophylaxis, and treatment.

We also searched the conference proceedings of the American Society of Clinical Oncology (ASCO, starting with its first volume, 1982 up to 2015) and of the American Society of Hematology (ASH, starting with its 2003 issue up to 2015). We also searched in the national cancer institute database and also in the guidelines from the European Society of Medical Oncology, National Institute of health and Care Excellence, the American College of Chest Physicians, and the European Society of Cardiology.

We reviewed the reference lists of included papers, relevant papers, and related systematic reviews.

We used the ‘related citation’ feature in PubMed to identify additional papers. We also searched ClinicalTrials.gov (clinicaltrials.gov/) for ongoing studies.

### Pathophysiology: how can we explain the frequency of VTE in patients with cancer?

The pathophysiology of thrombosis associated to cancer is complex and not entirely understood. Patients with cancer have a prothrombotic state resulting from the synergic activity of factors involved in the so-called Virchow’s triad: stasis of the blood caused by bed rest or by the tumor compression; vascular injury caused by intravasation of cancer cells, drugs, or therapeutic devices; and blood hypercoagulability is due to the release of cancer cell procoagulant factors, which affect the hemostasis process, including platelet functions and clotting cascade.

Key roles in pathophysiology are played by tissue factor (TF), inflammatory cytokines, and platelets.

TF is a transmembrane glycoprotein that has been identified to be the most likely candidate to explain procoagulant activity in cancer patients. Zwicker et al. evaluated the validity of the hypothesis assuming that the rise in circulating TF-bearing microparticles was associated with increased risks of VTE in cancer patients [19]. As results, TF-bearing microparticles were found elevated in 60% of cancer patients with VTE and in 27% of those without VTE, hence predicting an increased risk of VTE by fourfold

Injured endothelial and cancer cells expose TF on their membrane; TF is a receptor for the circulating coagulation factor VII, a serine protease that initiates the blood coagulation cascade, leading to an activation of other serine proteases: coagulation factors X, IX, VIII, V, and thrombin.

Circulating fibrinogen is then conversed into fibrin monomer, which polymerizes, and forms the fibrin-gel matrix. This matrix acts as a net, trapping platelets into a clot that contributes to the tissue repair [20,21]. At last, fibrinolytic enzymes, mostly plasmin, remove the clot, through the action of urokinase-type plasminogen activator (uPA) or tissue-type plasminogen activator (tPA).

In patients with cancer, fibrinolysis is counteracted by plasminogen-activator inhibitors (PAI) 1 and 2 that are particularly activated by cancer cells, resulting in enhanced chances of developing VTE [22].

Prostacyclin and thromboxane are also released from injured endothelial and cancer cells; they modulate platelet adhesion and aggregation [20]. These molecules are synthesized from arachidonic acid through a multistep process involving cyclo-oxygenases 1 (COX-1) and 2 (COX-2) [23].

Platelets also involved in cancer progression and metastasis have been identified to play a role in the hypercoagulable state of cancer [24]. In fact, activated platelets favor the adhesion of tumor cells to endothelial cells (EC) leading to their migration through the vessel wall by the release of heparanase activity. The platelets’ role in cancer progression is explained by their ability to protect tumor cells from innate immune cells [25]. Platelets’ contribution in tumor growth has been evaluated in many studies; Nierodzik et al. demonstrated in their study that experimental blockade of key platelet receptors, such as GP1b/IX/V, GPIIb/IIIa, and GPVI is associated to a decrease lung colonization of cancer cells, suggesting attenuation of the metastatic process [26]. Some recently reported data reported an increased survival with the use of aspirin in combination with surgical treatment of non-small cell lung cancer and colorectal cancer [27,28].

**Figure 1:**
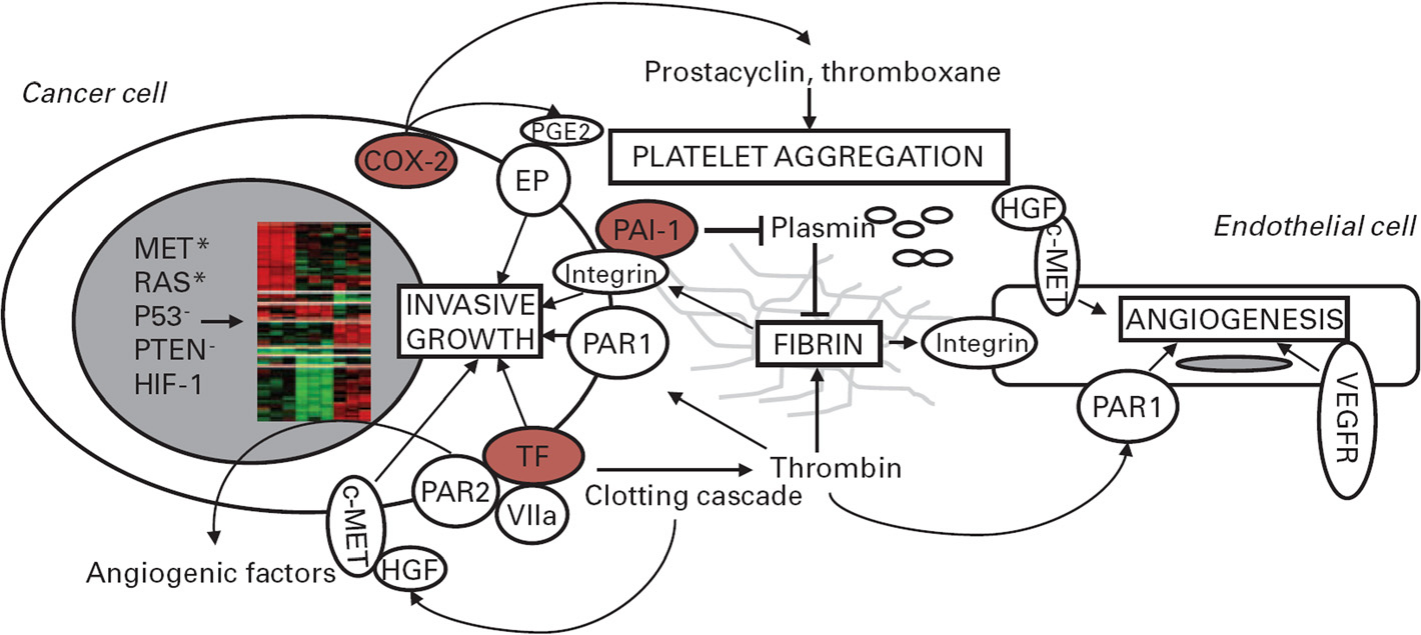
Hemostasis genes promote tumor progression. Activated oncogenes (MET*, RAS*), hypoxia-inducible factor-1 (HIF-1), and loss of tumor suppressor genes (PTEN-, P53-) induce transcriptional programs (nuclear heatmap) including tissue factor (TF), cyclo-oxygenase 2 (COX-2), and plasminogen-activator inhibitor 1 (PAI-1) upregulation. These, in turn, promote hemostasis activation and fibrin deposition. Fibrin forms a provisional matrix that favors angiogenesis and supports integrin-mediated cell adhesion and migration. Coagulation proteases activate hepatocyte growth factor (HGF), and thus the receptor encoded by the *MET* proto-oncogene (c-*MET*), which is expressed by endothelial and cancer cells. TF and thrombin generated by the coagulation cascade activate cell surface receptors (protease-activated receptors [PAR]-1 and −2). COX-2 catalyzes the synthesis of prostacyclin and thromboxane, which modulate platelet aggregation, and prostaglandin E2 (PGE2). The latter binds cell surface E-series prostaglandin receptors (EP). Besides inhibiting plasmin and fibrin degradation, PAI-1 promotes integrin recycling. MET, TF, PARs, EP, vascular endothelial growth factor receptor (VEGFR), and integrins cooperate in regulating cancer cell invasive growth and angiogenesis [23]

Figure 1 illustrates the contribution of different agents involved in the pathophysiology of VTE.

Cancer procoagulant (CP) is a cysteine protease only expressed by malignant cells and amniotic tissue. It has the particularity to directly activate factor X in the absence of factor VIIa [19]. CP has also been proven to stimulate blood platelet adhesion in a mechanism similar to thrombin and also induces platelet activation [19]. However, in the light of the available evidence, the precise role of CP in cancer-associated thrombosis remains not clearly defined.

Vascular injury also found to be involved in the prothrombotic state in patients with cancer is mostly caused by the treatment prescribed to this specific population. Chemotherapy for example is known to be associated with a 4.5- to 6-fold greater risk of thrombosis depending on the drug used [29]. Cisplatin and 5-fluorouracil, infused for the treatment of gastrointestinal tract, cervix, lung, and other cancers, are thrombogenic [30,31]. Also asparginase, used for the treatment of acute lymphoblastic leukemia, inhibits protein synthesis and leads to decreased levels of anticoagulant factors leading to an increased risk of VTE. Of the newer anticancer agents, the immunomodulatory drugs (IMiDss, thalidomide, and its analogues) carry a significant VTE risk [32,33].

A novel hypothesis has been recently suggested to explain the role of chemotherapy in cancer-associated thrombosis. It mostly includes DNA release from injured cells [34-37]; platelets are in fact trapped by intravascular DNA - in form of brands - resulting in a hypercoagulant state. (Table 1 illustrates possible mechanisms of VTE depending on chemotherapy drug.)

**Table 1:** Anticancer agents and possible mechanisms for VTE [36,37]

Anticancer or supportive agent	Presumed pathomechanism
Fluorinated pyrimidines (5 fluorouracil, capecitabine, tegafur-uracil, S1)	Vasospasm, arterial, and venous thrombosis
Cisplatin	Endothelial damage, Raynaud’s phenomenon, thrombosis (often combined with dexamethasone as an antiemetic)
L-asparaginase	Alters plasma levels of procoagulants and anticoagulants (AT III, protein C, protein S)
Tamoxifen	Alters plasma levels of coagulation factors
Dexamethasone	Alters plasma levels of coagulation factors
Erythropoiesis-stimulating agents	Alters plasma levels of coagulation factors, increased tissue factor expression
ImiDs (thalidomide, lenalidomide, etc.)	Endothelial damage, altered plasma levels of F. VIII, von Willebrand factor

Vessel wall damage can also be caused by extrinsic vascular compression, either by cancer-associated regional bulky lymphadenopathy or by the use of central venous access device for chemotherapy infusion [36,37].

Finally, there is the vessel stasis explained by the longer hospital stay in cancer patients. Heit et al. [38] reported nearly an increase of 22- and 8-fold, respectively, in the risk of developing VTE in patients hospitalized or confined to a nursing home with and without recent surgery.

Other risk factors for VTE are related to patient; age, for example (>60 years), is associated with a higher risk of VTE, and obesity and history of anterior VTE have also been reported to increase risks of VTE [39].

Some factors are related to the cancer itself such as the tumor’s site. Pancreatic cancer is considered to be on the top of solid tumors with high risk of VTE. Increased risk for VTE has also been noted in certain hematologic malignancies, such as lymphoma, acute leukemia, and multiple myeloma [1,40,41].

Histological type and tumor stage have also been defined as risk factors of VTE. Adenocarcinomas are associated with higher risks of VTE than squamous cell carcinomas [42], so is advanced stage as reported by Blom et al. that found an adjusted odds ratio of 19.8 for VTE risk in solid tumor cancer patients with distant metastases [43].

### VTE risk assessment in patients with cancer

According to the pathophysiology described above, VTE risk factors can be grouped in three general categories: patient-related factors, cancer-related factors, and treatment-related factors.

Predictive models have been established to assess the probability of developing VTE according to risk factors. The ‘Khorana Score’ for example, has been conceived to estimate the risk of VTE in ambulatory cancer patients receiving chemotherapy; it includes five predictive variables, cancer site, platelet count, hemoglobin level (or the use of erythropoiesis-stimulating agents), leukocyte count, and body mass index [38] (Tables 2 and 3). This model has the advantage to be simple and it uses readily available data [39-42].

**Table 2:** Risk factors for VTE in cancer patients

	Risk factors for VTE in cancer patients
Cancer-related factors	Tumor site
	Tumor’s histological type
	Tumor stage
	Tumor grade
	Initial period after diagnosis
Treatment-related factors	Surgery
	Radiotherapy
	Chemotherapy
	Antiangiogenic drugs
	Immunomodulatory drugs
	Hormonal therapy
	Therapy with erythropoiesis stimulating agents
	Blood transfusion
	Central lines
Patient-related factors	Age
	Weight, BMI
	Mobility
	Comorbidities
	Sepsis
	Compliance with prophylaxis
Other risk factors	Leukocyte count
	Platelet count
	Anemia
	Thrombophilia

**Table 3:** Predictive KHORANA model for chemotherapy-associated VTE in ambulatory cancer patients [38]

Risk factors	Number
Cancer-related risk factors	
Site of cancer and tumor histotype	2
Very high risk (stomach adenocarcinoma, pancreas	1
adenocarcinoma)	
High risk (lung, lymphoma, gynecological, bladder,	
testicular)	1
Hematological risk factors	1
Prechemotherapy platelet count ±350,000/l	1
Hemoglobin <10 g/dl or use of ESA growth factors	
Prechemotherapy leukocyte count >11 000/l	1
Patient-related risk factor	
Body mass index ±35 kg/m^2^	

Other predictive scores are under evaluation as an example PROTECHT Score’ adds platinum and gemcitabine-based chemotherapy to the predictive variables already taken into account in the Khorana model [43]. The ‘Ay Score’ adds D-dimer and soluble p-selectin as additional discriminatory risk factors for VTE in ambulatory cancer patients; however, its principal disadvantage is that the p-selectin is still a research marker and is not readily available in most laboratories [44]. Finally, there is the ‘Myeloma Working Group Score’ that is only valid for multiple myeloma patients [45].

The principal criticism for these scores is that they are derived from ambulatory patients receiving chemotherapy and concerns mostly patients with solid tumors and with a good performance status.

Validity of these scores to assess the risk for VTE in patients with poor performance status and those who are being treated with targeted therapies rather than ‘classical’ chemotherapy is not clear. Moreover, these predictive models indentify only high-risk patient which is not sufficient as VTE occurs more often in low-risk patient [43-48].

Despite these limitations, predictive models help physicians each day to define right candidates for prophylaxis. In fact, American Society of Clinical Oncology (ASCO) recommends that outpatient candidates for chemotherapy should be scored according to the Khorana model or other validated scores at the time of chemotherapy initiation and periodically thereafter [8]. The National Comprehensive Cancer Network (NCCN), the European Society of Medical Oncology (ESMO), and the National Institute of Health and Care Excellence guidelines (NICE) also include the Khorana Score along with other validated scores as an option to guide the decision-making regarding prophylaxis outpatients receiving chemotherapy guidelines [9-12]. However, neither the American College of Chest Physicians (ACCP) nor the European Society of Cardiology (ESC)uses those predictive models to indicate prophylaxis treatment [13,14].

### Diagnosis and evaluation of VTE in cancer patients

Like non-cancer patients, classical symptoms of lower extremity deep venous thrombosis (DVT) include pain, swelling, redness, warmness, and engorged superficial veins. In the prospective, multicenter registry (MASTER) of patients with VTE, the most common presenting symptoms of DVT were extremity edema, pain, and erythema observed in 80%, 75%, and 26% of patients with DVT, respectively [49]. However, many cancer patients with VTE do not have evident symptoms at presentation as their signs might be masked by the underlying malignancy.

As to pulmonary embolism (PE), the classic clinical signs include unexplained shortness of breath, chest pain, tachycardia, apprehension, tachypnea, syncope, and hypoxia The clinical presentation of PE can range from stable hemodynamics to cardiogenic shock. According to the MASTER registry, the most common presenting symptoms of PE were dyspnea, pain, and tachypnea, which were present in 85%, 40%, and 29% of patients with PE, respectively [49].

Clinical predictive models such as the Wells criteria have been evaluated and were proven useful in the diagnosis of VTE [50,51]. In patients with cancer, it is unclear whether this scoring system is as effective [52]. On one hand, evaluation of these models included only a minority of patients with cancer, and on the other hand, scoring 1 point is already given because of the malignancy.

D-dimer testing has been largely used as a diagnostic tool in non-cancer patients; it has a very good negative predictive value. In patients with cancer, D-dimer level could be elevated due to intravascular devices or coagulation activation by the tumor. It has been noted that the number of false positive D-dimer assays was threefold higher in cancer patients when compared to non-cancer patients [53]. In a large prospective study, D-dimer levels were high in cancer patients with suspected DVT while radiologic testing excluded the diagnosis of VTE [54]. Accordingly, most of the available guidelines do not suggest D-dimer testing for the diagnosis of VTE among patients with cancer [10-13], while some others consider a negative D-dimer test to have the same diagnostic value in cancer patient as in non-cancer patients however, cutoff level to 700 mg/L seems more interesting in this population [14].

Duplex utrasonography remains the number one choice for the diagnosis of lower extremity venous thrombosis it allows both an analysis of venous compressibility and Doppler imaging of venous blood flow [55,56]. Advantages of ultrasonography, include its accuracy in the diagnosis of DVT in femoral and popliteal veins without intravenous contrast agent, its ability to be done at the bedside, and, above all, its lower cost [56,57]. Its main inconvenient is that its results are operator-dependent [58].

Other imaging modalities are reserved for specific situations and can be performed in cases of negative or indeterminate ultrasound results. Magnetic resonance imaging (MRI) as an example is commonly used in these situations, and it is also specific in the evaluation of the pelvic, iliac veins, and vena cava [55,59,60]. The main disadvantages of MRI are its higher cost, longer imaging time duration, and its limited availability in some practice settings [59].

### VTE prophylaxis

#### Prophylaxis in hospitalized cancer patients

##### In medical cancer patient

To date, there has been no study evaluating the benefit-risk ratio of thromboprophylaxis conceived exclusively for hospitalized medical cancer patients.

Five randomized clinical trials including both cancer and non-cancer patients addressed this question. Three of them compared low molecular weight heparin (LMWH) with placebo in hospitalized patients with reduced mobility(5% to 15% of cancer patients) [61-63], and the other two compared LMWH to unfractionned heparin, all but one of these were double-blind [64,65].

All of these studies concluded that LMWH, unfractionned heparin (UFH), and fondaparinux were superior to placebo in preventing VTE, with non-significant increased bleeding risk [61-65].

Consequently, current guidelines recommend prophylaxis for hospitalized medical cancer patients [8-13].

LMWH, fondaparinux, or UFH can equally be used [48-50,61-65]. There is a trend toward to prefer LMWH and fondaparinux over UFH because of their ease of administration. Once started, prophylaxis should be continued till full recovery or until discharge from hospital [48-50,65].

In contrast with their high risk of VTE, cancer patients appear to have a high bleeding risk when compared to the general population. Therefore, attention should be paid regarding contraindications and risks associated to anticoagulation (Table 4).

**Table 4:** Contraindications to anticoagulation treatment

	Contraindications
Absolute contraindications	Active major, serious, or potentially life-threatening bleeding not reversible with medical or surgical intervention, including but not limited to any active bleeding in a critical site (i.e., intracranial, pericardial, retroperitoneal, intraocular, intra-articular, intraspinal) [10-12^a^]
	-Active bleeding (major): more than 2 units transfused in 24 h, chronic [11,12^a^]
	-Severe, uncontrolled malignant hypertension [10,12^a^]
	-Severe, uncompensated coagulopathy (e.g., liver failure) [10]
	-Severe platelet dysfunction or inherited bleeding disorder [10-12^a^]
	-Persistent, severe thrombocytopenia (20,000/L) [10]
	-Surgery or invasive procedure, including but not limited to lumbar puncture, spinal anesthesia, and epidural catheter placement [10-12^a^]
Relative contraindications	-Intracranial or spinal lesion at high risk for bleeding [10-12]
	-Active peptic or other GI ulceration at high risk of bleeding [10,12]
	-Active but non-life-threatening bleeding (e.g., trace hematuria) [10]
	-Intracranial or CNS bleeding within past 4 weeks [10]
	-Major surgery or serious bleeding within past 2 weeks [10-12]
	-Persistent thrombocytopenia (50,000/L) [10-12]
	-Chronic, clinically significant measurable bleeding >48 h [11]
	-High risk for falls (head trauma) [11]

^a^For ESMO guidelines, all the contraindications are referred as relative

##### In surgical cancer patients

It is now clearly established that patients with cancer undergoing surgery are at higher risk of developing VTE when compared to non-cancer patients [2,3].

Also in this setting, trials evaluating prophylaxis in patients undergoing surgery concerned both cancer and non-cancer patients. Only one randomized controlled study concerned only cancer patients. It included 99 Indian cancer patients undergoing colorectal surgery and compared LMWH for 6 days with no prophylaxis with no difference between the two groups [66].

Three meta-analyses compared LMWH or UFH to placebo; one was conducted in general surgery patients [67] and the two others concerned patients undergoing gynecologic surgery [68,69]. The main result was the superiority of LMWH and UFH over placebo in terms of preventing postoperative VTE. Only one meta-analysis showed a higher rate of bleeding associated with LMWH [67].

The question that remains is the choice of the optimal drug for prophylaxis. Three randomized double-blind studies tried to answer this question and compared LMWH with UFH in the prevention of VTE in surgical patients two of them included exclusively cancer patients [70,71] and one included 35.2% of cancer patients undergoing colorectal surgery [72]. Results showed no difference in terms of effectiveness between LMWH and UFH. Three other meta-analyses confirmed these results and reported that UFH given three times a day is as effective as LMWH given once a day [67,69,73]. In terms of bleeding, both regimens showed the same results.

Concerning the optimal dose, only one double-blind trial was conducted it compared subcutaneous 2,500 anti-Xa IU and 5,000 anti-Xa IU of Dalteparin administered for 8 days to 1,375 patients undergoing major elective abdominal surgery, and results showed that higher doses were more effective [74].

Giving these results, current guidelines have made specific recommendations concerning postoperative VTE prevention [8-13] (Table 5). LMWH or UFH are recommended for VTE prevention in the postoperative setting. Mechanical methods such as pneumatic calf compression may be added to pharmacological prophylaxis but should not be used as monotherapy unless pharmacological prophylaxis is contraindicated.

**Table 5:** Summary of international guidelines regarding thromboprophylaxis in hospitalized cancer patients

	Medical patient	Surgical patient
NCCN Guidelines (2014) [9]	-Prophylactic anticoagulation therapy(category 1)	-Prophylactic anticoagulation therapy (category 1)
	± Intermittent pneumatic venous compression device (IPC)	
	± Graduated compression stockings (GCS)	± Intermittent pneumatic venous compression device (IPC)
		± Graduated compression stockings (GCS)
		-Out-of-hospital primary VTE prophylaxis is recommended for up to 4 weeks postoperation (particularly for high-risk abdominal or pelvic cancer surgery patients)
		-Mechanical methods are not recommended as monotherapy except when pharmacological methods are contraindicated.
ASCO Guidelines (2015) [8]	1. Hospitalized patients who have active malignancy with acute medical illness or reduced mobility should receive pharmacologic thromboprophylaxis in the absence of bleeding or other contraindications.	1. All patients with malignant disease undergoing major surgical intervention should be considered for pharmacologic thromboprophylaxis with either UFH or LMWH unless contraindicated because of active bleeding or high bleeding risk.
	Evidence: strong	Evidence: strong
	2. Hospitalized patients who have active malignancy without additional risk factors may be considered for pharmacologic thromboprophylaxis in the absence of bleeding or other contraindications.	2. Prophylaxis should be commenced preoperatively. Evidence: moderate
	Evidence: moderate	3. Mechanical methods may be added to pharmacologic thromboprophylaxis but should not be used as monotherapy for VTE prevention unless pharmacologic methods are contraindicated because of active bleeding or high bleeding risk.
		Evidence: moderate
		4. A combined regimen of pharmacologic and mechanical prophylaxis may improve efficacy, especially in the highest risk patients.
		Evidence: moderate
		5. Pharmacologic thromboprophylaxis for patients undergoing major surgery for cancer should be continued for at least 7 to 10 days. Extended prophylaxis with LMWH for up to 4 weeks postoperatively should be considered for patients undergoing major abdominal or pelvic surgery for cancer who have high-risk features such as restricted mobility, obesity, history of VTE, or with additional risk factors. In lower-risk surgical settings, the decision on appropriate duration of thromboprophylaxis should be made on a case-by-case basis considering the individual patient.
	Recommendation type, strength: evidence based, strong	
	3. Data are inadequate to support routine thromboprophylaxis in patients admitted for minor procedures or short chemotherapy infusion or in patients undergoing stem-cell/bone marrow transplantation.	
ESMO Guidelines (2011) [10]	Prophylaxis with UFH, LMWH or fondaparinux is recommended [I, A].	In cancer patients undergoing major cancer surgery:
		Prophylaxis with LMWHs or UFH is recommended. Mechanical methods such as pneumatic calf compression may be added to pharmacological prophylaxis but should not be used as monotherapy unless pharmacological prophylaxis is contraindicated because of active bleeding [I,A].
		Cancer patients undergoing elective major abdominal or pelvic surgery:
		Should receive in hospital and postdischarge prophylaxis with LMWH for up to 1 month after surgery [I, A].
ISTH Guidelines (2013) [11]	1. We recommend prophylaxis with LMWH, UFH or fondaparinux in hospitalized medical patients with cancer and reduced mobility (grade 1B).	1. Use of LMWH once a day or a low dose of UFH three times a day is recommended to prevent postoperative VTE in cancer patients; pharmacological prophylaxis should be started 12 to 2 h preoperatively and continued for at least 7 to 10 days; there are no data allowing conclusions regarding the superiority of one type of LMWH over another (grade 1A).
		Values and preferences: LMWH once a day is more convenient
		2. There is no evidence to support fondaparinux as an alternative to LMWH for the prophylaxis of postoperative VTE in cancer patients (grade 2C).
		Values and preferences: similar
		3. Use of the highest prophylactic dose of LMWH to prevent postoperative VTE in cancer patients is recommended (grade 1A).
		Values and preferences: equal
		4. Extended prophylaxis (4 weeks) to prevent postoperative VTE after major laparotomy in cancer patients may be indicated in patients with a high VTE risk and low bleeding risk (grade 2B).
		Values and preferences: longer duration of injections
		5. The use of LMWH for the prevention of VTE in cancer patients undergoing laparoscopic surgery may be recommended in the same way as for laparotomy [best clinical practice, based on a balance between desirable and undesirable effects indicating an increased bleeding risk].
		Values and preferences: daily injections
		Costs: In some countries, the price of LMWH may influence the choice.
		6. Mechanical methods are not recommended as monotherapy except when pharmacological methods are contraindicated (grade 2C).
		Values and preferences: no injection
ACCP guidelines [13]		1. For high-VTE-risk patients undergoing abdominal or pelvic surgery for cancer who are not otherwise at high risk for major bleeding complications, extended duration pharmacologic prophylaxis (4 weeks) with LMWH over limited-duration prophylaxis is recommended (grade 1B).

Extended prophylaxis is strongly recommended especially for patients undergoing major abdominal or pelvic surgery [8-13]. This recommendation is based on the results of two randomized trials and one meta-analysis that showed better outcomes with extended postoperative prophylaxis after major laparotomy surgery [75,76]

#### Prophylaxis in ambulatory cancer patients

Nowadays, most cancer patients are being treated as outpatients as an effort in shortening hospital stays (Tables 6 and 7).

**Table 6:** Summary of international guidelines related to thromboprophylaxis in ambulatory cancer patients

	Summary of international guidelines
NCCN (2014) [9]	1. Multiple myeloma patients receiving thalidomide or lenalidomide:
	-High risk: Recommend anticoagulant VTE prophylaxis
	-Low risk: Recommend aspirin
	2. Other outpatient settings:
	No routine VTE prophylaxis recommended outside of a clinical trial setting
ASCO (2015) [8]	1. Routine pharmacologic thromboprophylaxis is not recommended in cancer outpatients.
	Evidence: moderate.
	2. Based on limited RCT data, clinicians may consider LMWH prophylaxis on a case-by-case basis in highly selected outpatients with solid tumors receiving chemotherapy.
	Consideration of such therapy should be accompanied by a discussion with the patient about the uncertainty concerning benefits and harms as well as dose and duration of prophylaxis in this setting.
	Evidence: moderate
	3. Patients with multiple myeloma receiving thalidomide- or lenalidomide-based regimens with chemotherapy and/or dexamethasone should receive pharmacologic thromboprophylaxis with either aspirin or LMWH for lower-risk patients and LMWH for higher-risk patients.
ESMO (2011) [10]	1. Extensive, routine prophylaxis for advanced cancer patients receiving chemotherapy is not recommended, but may be considered in high-risk ambulatory cancer patients [II, C].
	2. Consider LMWH, aspirin or adjusted-dose warfarin (INR 1.5) in myeloma patients receiving thalidomide plus dexamethasone or thalidomide plus chemotherapy [II, B].
ISTH (2013) [12]	1. For children with ALL treated with L-asparaginase, depending on local policy and individual patient characteristics (platelet count, kidney function, fibrinogen and antithrombin III levels, etc.), prophylaxis may be considered in some patients; the same therapeutic option can be considered for adults [best clinical practice, based on evidence of very low quality].
	2. In patients receiving chemotherapy, prophylaxis is not recommended routinely [grade 1B].
	3. Primary pharmacological prophylaxis of VTE may be indicated in patients with locally advanced or metastatic pancreatic cancer treated with chemotherapy and having a low bleeding risk [grade 1B].
ACCP [13]	1. In outpatients with cancer who have no additional risk factors for VTE, routine prophylaxis with LMWH or LDUH is not suggested (grade 2B) and the prophylactic use of VKAs is not recommended (grade 1B).
	2. In outpatients with cancer and indwelling central venous catheters, routine prophylaxis with LMWH or LDUH is not suggested (grade 2B), neither is the prophylactic use of VKAs (grade 2C).

**Table 7:** Dosing regimens for prophylaxis and treatment of VTE in patients with cancer [8]

	Dose
Pharmacologic (anticoagulant) prophylaxis	
Hospitalized medical patients	
Unfractionated heparin	5,000 U once every 8 h sc
Dalteparin	5,000 U once daily
Enoxaparin	40 mg once daily
Fondaparinux	2.5 mg once daily
Surgical patients	
Unfractionated heparin	5,000 U 2 to 4 h preoperatively and once every 8 h sc thereafter or 5,000 U 10 to 12 h preoperatively and 5,000 U once daily thereafter
Dalteparin	2,500 U 2 to 4 h preoperatively and 5,000 U once daily thereafter or 5,000 U 10 to 12 h preoperatively and 5,000 U once daily thereafter
Enoxaparin	20 mg 2 to 4 h preoperatively and 40 mg once daily thereafter or 40 mg 10 to 12 h preoperatively and 40 mg once daily thereafter
Fondaparinux	2.5 mg beginning 6 to 8 h postoperatively
Treatment of established VTE	
Initial	
Unfractionated heparin	80 U/kg IV bolus, then 18 U/kg per hour IV
Dalteparin	100 U/kg once every 12 h; 200 U/kg once daily
Enoxaparin	1 mg/kg once every 12 h; 1.5 mg/kg once daily
Tinzaparin	175 U/kg once per day
Fondaparinux	50 kg, 5.0 mg once daily; 50 to 100 kg, 7.5 mg once daily; 100 kg, 10 mg once daily
Long term	
Dalteparin	200 U/kg once daily for 1 month, then 150 U/kg once daily
Enoxaparinijk	1.5 mg/kg once daily; 1 mg/kg once every 12 h
Tinzaparin	175 U/kg once daily
Warfarin	Adjust dose to maintain INR 2 to 3

While recommendations for VTE prevention among hospitalized patients are clearly established, benefice of VTE prophylaxis for cancer outpatients is not well-defined.

To address this question, two prospective randomized studies compared LMWH with placebo [46,77], PROTECHT (nadroparin, 1,150 patients) and SAVE-ONCO (semuloparin, 3,212 patients). Both of these studies reported reductions in symptomatic DVT (from 2% to 4% to 1% to 2%) and PE (from 0.8% to 0.9% to 0.2% to 0.5%) without increasing the risks of bleeding. Three other randomized double-blind trials along with an analysis of pooled data from two other randomized double-blind studies compared LMWH to placebo [77-84]. Main results were the decrease of VTE rate in patients with locally advanced or metastatic pancreatic and lung cancers when LMWH primary prophylaxis was employed. There was a trend toward bleeding increase especially in the context of thrombocytopenia.

According to available data, NCCN panel along with ESMO, ACCP, and the International Society of thrombosis and Haemostasis (ISTH) suggest to evaluate the risks and benefits of thromboprophylaxis in ambulatory cancer patients. Predictive models such as the Khorana model or other validated scores should be used to determine patients that will benefit most from prophylaxis [8-12]. However, specific considerations are accorded to lung and pancreatic cancer, especially in ESMO and ISTH guidelines where prophylaxis is systematically recommended for these localizations [12,14].

For patient with multiple myeloma, the International Myeloma Working Group recommends prophylaxis with either LMWH or dose-adjusted warfarin for patients receiving lenalidomide- or thalidomide-based combination regimens and also for patients with two or more individual or disease-related risk factors as defined by the group [41].

### Treatment of established VTE

Treatment of VTE in general population consists of an initial treatment with a rapid acting parenteral anticoagulation with LWMH or UFH or fondaparinux overlapping with and followed by an oral vitamin K antagonist (VKA) (Tables 7 and 8).

**Table 8:** Summary of available international guidelines concerning the treatment of established VTE

	Initial treatment	Early maintenance and long term treatment
NCCN (2014) [9]	LMWH is recommended for the initial treatment of established VTE in cancer patients. (Category 1)	1. LMWH (category 1) is preferred for the first 6 months as monotherapy without warfarin in patients with proximal DVT or PE and prevention of recurrent VTE in patients with advanced or metastatic cancer.
		2. If warfarin is selected for chronic anticoagulation (category 2b), initiate warfarin concurrently with the parenteral agent used for acute therapy and continue both therapies for at least 5 days and until the INR 2 for 24 h.
		During the transition to warfarin monotherapy, the INR should be measured at least twice weekly. Once the patient is on warfarin alone, the INR should be measured initially at least once weekly. Once the patient is on a stable dose of warfarin with an INR between 2 and 3, INR testing can be gradually decreased to a frequency no less than once monthly.
ESMO (2011) [10]	LMWH is recommended for the initial treatment of established VTE in cancer patients.	Long-term treatment for 6 months with 75% to
		80% (that is, 150 U/kg once daily) of the initial dose of LMWH is safe and more effective than treatment with a VKA. This schedule is recommended for Long term anticoagulant therapy in cancer patients [I, A].
ISTH (2013) [12]	1. LMWH is recommended for the initial treatment of established VTE in cancer patients [grade 1B].	1. LMWHs are preferred over VKA for the early maintenance treatment (10 days to 3 months) and long-term treatment (beyond 3 months) of VTE in cancer patients [grade 1A].
	Values and preferences: LMWHs are easier to use than UFH.	
	2. Fondaparinux and UFH can be also used for the initial treatment of established VTE in cancer patients [grade 2D].	Values and preferences: daily subcutaneous injection may represent a burden for patients.
		2. Idraparinux is not recommended for the early maintenance treatment (10 days to 3 months) and the long-term treatment (beyond 3 months) of VTE in cancer patients; idraparinux is currently not available on the market [grade 2C]. Values and preferences: idraparinux once weekly is easier to use than UFH or LMWH.
	Values and preferences: fondaparinux is easier to use than UFH.	
		3. LMWH should be used for a minimum of 3 months to treat established VTE in cancer patients; however, patients were treated for 6 months in the largest study in this setting [grade 1A].
		Values and preferences: daily subcutaneous injection may represent a burden for patients.
		4. After 3 to 6 months, termination or continuation of anticoagulation (LMWH or VKA) should be based on individual evaluation of the benefit-risk ratio, tolerability, patients’ preference, and cancer activity [best clinical practice, in the absence of data].
European society of cardiology (ESC) [14]	LMWH should be administered in the acute phase	1. LMWH administered in the acute phase Should be continued over the first 3 to 6 months and is considered as first-line therapy.
		2. Chronic anticoagulation (beyond 3 months) may consist of continuation of LMWH, transition to VKA, or discontinuation of anticoagulation. The decisions should be made on a case-by-case basis.
		3. Treatment of cancer-related VTE with fondaparinux and the new oral anticoagulants is limited.
American College of Chest Physician (ACCP) [13]		1. In patients with DVT of the leg and cancer, LMWH is suggested over VKA therapy (grade 2B).
		2. In patients with DVT and cancer who are not treated with LMWH, VKA is suggested over dabigatran or rivaroxaban for long-term therapy (grade 2B).
		3. In patients with DVT of the leg and active cancer, if the risk of bleeding is not high, extended anticoagulant therapy over 3 months of therapy is recommended (grade 1B), and if there is a high bleeding risk, extended anticoagulant therapy is suggested (grade 2B).
		4. In patients with PE and cancer, the treatment is as suggested in patient with DVT.

Available data suggest that this regimen cannot be applied for cancer patients, especially because of the higher risks of bleeding and recurrence in this particular population.

#### Initial treatment

Initial treatment is defined as the first 10 days of anticoagulation treatment.

In our review, we found only retrospective studies in cancer patients evaluating LWMH or UFH followed by VKA. Five randomized studies concerned LMWH in association with VKA, and six others concerned UFH with VKA. Overall, recurrence rate was not negligible, and it reached 6.7% to 16.9% with LMWH and 11% to 38% with UFH; the two drugs were overlapped and followed by an oral vitamin K. Major bleeding was also evaluated up to 10 months of follow-up, and both treatments were associated with high rates of bleeding [85-88].

Authors concluded that either LMWH or UFH combined to VKA is associated with high rates of recurrence and bleeding [88]. Therefore, early relay with VKA should not be advised to this particular population.

As to the choice of the optimal rapid acting parenteral anticoagulant, we found two meta-analyses of trials comparing LMWH and UFH among cancer patients; no statistical difference was found in the rates of recurrence and major bleeding between the two drugs [88,89]. The most interesting about these studies is the unexpected beneficial effect of LMWH on the risk of death; in the most recent meta-analysis among 801 cancer patients, the used of LMWH reduced the death rate from 18.9% to 13.1% with a relative of 0.71 [88].

As to fondaparinux, one randomized controlled trial compared fondaparinux and LMWH. In a *post hoc* analyses of cancer patients’ subgroup, rates of recurrence at 3 months and major bleeding were not different between the two groups [85,88].

According to these findings, current guidelines recommend either LMWH, UFH, or, in some cases, fondaparinux in the initial treatment of VTE [9-14].

#### Early maintenance and long-term treatment of established VTE

Early maintenance is defined as the period beyond the tenth day and up to the third month of anticoagulation. Long-term treatment is the period beyond the third month of anticoagulation.

Six randomized trials and five meta-analyses focused on long-term treatment of VTE [85-93].

Three of the randomized trials reported that in cancer patients, extended LMWH treatment was associated with less VTE recurrence without increasing bleeding risk [85-87]. The CANTHANOX study added that LMWH was not only more effective than VKA but also was associated with a reduced risk of major bleeding at 3 months (*P* = 0.04) [92].

As to the meta-analyses [89-93], all but one concluded that early maintenance and long-term treatment with LMWH decreased the VTE recurrence rate by 50% [89-93]. No increase in bleeding risk was found in the extended LMWH treatment arm [90-93].

As a conclusion, in cancer patients with VTE, early maintenance treatment (10 days to 3 months) and long-term treatment (beyond 3 months) with LMWH showed better outcomes in terms of VTE recurrence without majoring the risk of bleeding.

Current guidelines relied on these results to recommend long-term treatment for 6 months with 75% to 80% (that is, 150 U/kg once daily) of the initial dose of LMWH (Table 6 for dosing schedules). Experts consider this treatment schedule safe and more effective when compared to early relay with VKA [9-14].

### Inferior vena cava filter (VCF)

Inferior vena cave filters (VCFs) are used whenever contraindications to anticoagulation are present. Recurrent VTE despite adequate anticoagulant treatment is another indication for VCF [9-13]

Fourteen retrospective cohort studies concerned utilization of vena cava filters in cancer patients, and their results support the feasibility of placing the vena cava filter in cancer patients [94-104] technical precautions should however be taken especially in patient with metastatic stage.

### Idiopathic VTE and cancer screening

Asymptomatic cancers are not uncommon. Spontaneous VTE can be an alarm signal for underlying malignancy [105-113].

Screening for occult malignancy in patients with symptomatic idiopathic venous thromboembolism (SOMIT) is a prospective study performed in Italy to assess if an extensive screening program is necessary to identify early stage in order to improve treatment possibilities and diseases’ prognosis [114]. Results showed that extensive screening was able to detect most of the hidden malignancies with a high degree of sensitivity. However, it did not have any impact on overall survival that was the end point of the study.

Other studies succeeded the SOMIT study to address extensive screening in patient with spontaneous VTE, and some even considered PET scan [115]. To date, no study has reported a benefit in survival with extensive screening [116,117]. A Cochrane meta-analysis has started on November 2013 and will include all the available trials addressing this question; results are still awaited.

ESMO and the NICE are the two available guidelines that recommend screening programs for occult malignancy in patient with idiopathic VTE.

According to NICE guidelines, physical examination, chest X-ray, blood tests, and urinalysis should be performed. Further investigations are to be considered (abdomino-pelvic CT scan and mammogram for women) in all patients of 40 years and above with a first unprovoked DVT or PE who do not have symptoms of cancer based on initial investigation [13].

As to ESMO guidelines, patients should undergo physical examination, chest X-ray, occult fecal blood test, urological visit in men, and gynecological visit in women. More expensive examinations such as computed tomography (CT) scan, digestive endoscopy, or tumor markers should not be performed unless strong clinical suspicion of occult cancer is present [12].

### Special situations

#### Patients with brain tumors

Primary central nervous system (CNS) tumors are not very common; however, their incidence has been increasing over the last 30 years, especially in elderly persons [118]. Metastatic disease to the CNS occurs ten times more often than primary brain tumors. It is estimated that 20% to 40% of patients with systemic cancer will develop brain metastases [119].

The specificity of brain tumors is that paradoxically with their high thrombosis risk, they can be complicated by hemorrhagic transformation or tumor infiltration of the spinal cord with a potential risk for intra-spinal bleeding. Thereby, specific considerations have been accorded to this localization.

The principal conclusion that was drawn from the few studies that concerned VTE in patients with brain tumors is that brain tumor per se is not a contraindication to anticoagulation [120,121]. As to prophylaxis, in medical patients, benefits and risks have to be weighed individually using predictive scores such as the Khorana model to indicate treatment [11-14]. While in surgical patient, prophylaxis is recommended systematically [11-14]. As for other tumor sites, contraindications have to be evaluated before any treatment, and also, monitoring for brain bleeding should be performed [122]. In patients with established VTE, treatment with anticoagulant is recommended according to the schedule established for other localizations, and special attention should be paid to the risk of brain bleeding.

#### Catheter-related thromboses

In the last two decades, two open-label randomized clinical trials concluded that anticoagulant prophylaxis is beneficial in reducing VTE risks in patients with central venous catheter (CVC) [123,124].

However, recent studies do not support this conclusion. In fact, four recent randomized studies suggested that giving the low incidence of CVC-related VTE (3% to 4%), systematic prophylaxis was not justified [125-127].

Current guidelines do not recommend routine prophylaxis to prevent CVC-related VTE [9-13].

As to the treatment of established VTE, treatment is as described above. If the catheter is correctly positioned and functional with no signs of infection and still required for patient care, guidelines do not recommend removing the device. Otherwise, CVC should be removed and also in the case of VTE recurrence despite an adequate anticoagulation [9].

#### Other special situations

##### Patients with renal insufficiency

Dosage adjustments for renal failure are available and should be applied for each approved LMWHs’ treatment on a case-by-case basis and according each case creatinine clearance.

In patients with severe renal failure (creatinine clearance <30 mL min), UFH can be used on a case-by-case basis [9,14].

##### Patients with thrombocytopenia

Full doses of anticoagulant can be used for the treatment of established VTE if the platelet count is >50 G L. There is no evidence of a major risk of bleeding when platelet count is below 50 G L, and decisions on treatment and dosage should be made on a case-by-case basis with the utmost caution [8-14].

### Anticoagulant as a cancer treatment in patients without VTE

As described, there are many interactions between coagulation activation and tumor growth; blocking the clotting cascade with anticoagulant agents can lead to a disruption of the tumor proliferation process [128,129]. Accordingly, we can assume that anticoagulation may have some antitumor activity [130-133].

Few clinical trials and some limited case reports addressed this question [134-143]. While older reports suggested a beneficial effect of anticoagulation, the newer studies did not support this conclusion [144-147].

A Cochrane meta-analysis included nine RCTs enrolling 2,857 patients. Heparin, either unfractionated heparin or low molecular weight heparin, was evaluated in all of the included RCTs [148]. Authors concluded that heparin was associated with a significant reduction of death at 24 months but not at 12 months. Anticoagulation was also associated with a reduction in venous thromboembolism with no significant effect on major bleeding, minor bleeding, or quality of life (QoL).

Future research should further investigate the survival benefit of different types of anticoagulants in patients with different cancer types and stages of cancer. Decision to prescribe anticoagulation for cancer patients without VTE should balance benefits and risks and also integrate patient values and preferences [148].

### New oral anticoagulants (NOACs) in cancer patients

New oral anticoagulants (NOACs) are a new achievement in the management of thrombosis; they directly inhibit factor Xa or thrombin. These agents are very attractive as they can be taken orally, without the need of dose adjustment, they also do not have drug interactions, and moreover, they do not require monitoring. Dabigatran, a direct thrombin inhibitor, and rivaroxaban and apixaban, two direct factor Xa inhibitors, are the most developed agents.

In non-cancer patients, these drugs have proven their effectiveness in VTE prophylaxis in the postoperative setting also in stroke prevention in patients with non-valvular atrial fibrillation [149]. Moreover, they have been shown to be effective in the prevention of recurrent VTE. Rivaroxaban has in fact been approved as monotherapy in the treatment of DVT [149-151].

Studies evaluating NOACs in medical patients included small numbers of patients with cancer. No studies have specifically evaluated the treatment of cancer-associated VTE using these agents. Only a small phase 2 study evaluated the safety and tolerability of apixaban in patients with cancer. Authors reported low risk of major bleeding (2.2%) during 12 weeks of therapy in 125 patients with metastatic or advanced cancer without thrombosis [152].

In a subgroup analysis of thromboprophylaxis with rivaroxaban, a trend to less efficacy although not significant - was noted in the enoxaparin arm among patients with active cancer [153]. In another subgroup analysis of a rivaroxaban trial, the reported results were different; rivaroxaban was associated with a (non-significant) reduction of VTE and less bleeding. The main criticism of this study is that VKA was the comparator used which is not considered as the optimal choice for cancer patients [154].

Giving this limited data, current guidelines do not recommend the routine use of NOACs either in VTE prophylaxis or in the treatment of established VTE [8-13].

## Conclusions

In 2002, a survey among oncologists in northern England found that more than a quarter of oncologists do not recognize the thrombogenic effects of the treatments prescribed to patients with cancer. Other surveys have also made the conclusion that VTE in patients with cancer is being underestimated and thromboprophylaxis is rarely prescribed. Since, interest has been accorded to VTE in patients with cancer especially that higher rates of recurrence and major bleeding have been noted for this population.

Nowadays, guidelines have been established to improve the management and outcomes for cancer patients with VTE, and the question of VTE in cancer patient has become more clarified, especially regarding the prophylaxis. In fact, prophylactic anticoagulation therapy is recommended for all *inpatients* with a diagnosis of active cancer who do not have a contraindication to such therapy.

For *surgical outpatients*, extended prophylaxis (4 weeks) with LMWH is recommended over limited duration prophylaxis for patients undergoing abdominal or pelvic surgery who are not otherwise at high risk for major bleeding complications.

As to *medical outpatients*, prophylaxis is given after an evaluation of the benefits and risks of anticoagulation; predictive models such as the Khorana model might be used to select appropriate candidates for prophylaxis.

It is the time for us, physicians to change our clinical daily practice.

## Abbreviations

ACCP: American College of Chest Physicians
ASCO: American Society of Clinical Oncology
CNS: central nervous system
CVC: central venous catheter
DVT: deep venous thrombosis
ESC: European society of cardiology
ESMO: European Society of Medical Oncology
ISTH: International Society of thrombosis and Haemostasis
LMWH: low molecular weight heparin
NCCN: National Comprehensive Cancer Network
NICE: National Institute of health and Care Excellence guidelines
PE: pulmonary
SOMIT: Screening for Occult Malignancy in Patients with Symptomatic Idiopathic Venous Thromboembolism
UFH: unfractionned heparin
VTE: venous thromboembolism
